# Shifts in community composition and co-occurrence patterns of phyllosphere fungi inhabiting *Mussaenda shikokiana* along an elevation gradient

**DOI:** 10.7717/peerj.5767

**Published:** 2018-10-12

**Authors:** Xin Qian, Liang Chen, Xiaoming Guo, Dan He, Miaomiao Shi, Dianxiang Zhang

**Affiliations:** 1 Key Laboratory of Plant Resources Conservation and Sustainable Utilization, South China Botanical Garden, Chinese Academy of Sciences, Guangzhou, Guangdong, China; 2 University of Chinese Academy of Sciences, Beijing, China; 3 CAS Key Laboratory of Pathogenic Microbiology and Immunology, Institute of Microbiology, Chinese Academy of Sciences, Beijing, China; 4 Center for Ecological and Environmental Sciences, South China Botanical Garden, Chinese Academy of Sciences, Guangzhou, Guangdong, China

**Keywords:** Phyllosphere fungi, Biodiversity, Elevation gradient, Mussaenda shikokiana, High-throughput sequencing

## Abstract

The altitudinal effects on the distributions of phyllosphere fungal assemblages in conspecific plants remain poorly elucidated. To address this, phyllosphere fungal communities associated with *Mussaenda shikokiana* were investigated at four sites across a 350 m elevation gradient in a subtropical forest by employing Illumina metabarcoding of the fungal internal transcribed spacer 2 (ITS2) region. Our results demonstrated that phyllosphere fungal assemblages with a single host possessed high taxonomic diversity and multiple trophic guilds. OTU richness was significantly influenced by elevation. The elevation gradient also entailed distinct shifts in the community composition of phyllosphere fungi, which was significantly related to geographical distance and mean annual temperature (MAT). Additionally, comparison of phyllosphere fungal networks showed reduced connectivity with increasing elevation. Our data provide insights on the distribution and interactions of the phyllosphere fungal community associated with a single host along a short elevation gradient.

## Introduction

The fungal kingdom represents one of the largest and most diverse eukaryotic lineages with estimates of around 5.1 million extant species (Blackwell, 2011; Truong et al., 2017). Fungi function as important ecosystem components that govern global carbon cycling, plant pathology, development and reproduction (Tedersoo et al., 2014). Although fungi directly impact ecosystem functions, only a fraction of the total fungal species have been described (Truong et al., 2017). Therefore, it is essential to obtain adequate taxonomic and ecological knowledge of members of the fungal lineage to advance basic ecology and to recognize and predict the future consequences of environmental changes (Bryant et al., 2008; Geml et al., 2014).

The distribution pattern of species communities along altitudinal gradients is a fundamental theme of ecology and biogeography, mainly because altitudinal gradients are often characterized by different climatic conditions and complex changes in abiotic and/or biotic factors over short geographic distances (Bryant et al., 2008; Ren et al., 2018; Yao et al., 2017). Although there is a long history of studies that have been conducted on the elevational patterns of vascular plants and animals (Bryant et al., 2008), similar studies have only recently been made possible for microbial communities with the advent of culture-independent molecular techniques (Datlof et al., 2017; Lanzen et al., 2016; Wainwright et al., 2017; Zhang et al., 2018c). Several studies of the elevation patterns of fungal communities showed no consistent conclusions. In a tropical montane cloud forest, Looby, Maltz & Treseder (2016) documented increasing alpha-diversity of soil fungi and a significant shift in the community composition with increasing elevation. A similar elevational pattern was observed in soil fungal communities in the Italian Alps (Siles & Margesin, 2016). However, opposite patterns of species richness were found for ectomycorrhizal fungi in the Hyrcanian forests of northern Iran (Bahram et al., 2012) and for arbuscular mycorrhizal fungi in a Tibetan alpine grassland (Liu et al., 2015). In addition, a unimodal pattern, with a mid-elevation peak in diversity, was detected in ectomycorrhizal fungal communities on the northwest slopes of the Fuji mountains (Miyamoto et al., 2014). In a study conducted in the Cairn Gorm mountains in Scotland, Jarvis, Woodward & Taylor (2015) found that elevation did not influence ectomycorrhizal fungal richness of Scots pine, but strongly influenced community composition. Along gradients in the Changbai mountains in China, neither the species richness nor community composition of soil fungi were significantly correlated with elevation (Shen et al., 2014). These different distribution patterns may partly be explained by difference in environmental variables, including edaphic qualities (Geml et al., 2014; Jarvis, Woodward & Taylor, 2015; Shen et al., 2014; Siles & Margesin, 2016), mean annual temperature and precipitation (Bahram et al., 2012). However, most studies focused only on soil or root associated fungal assemblages, and few studies have investigated the altitudinal effects on the distribution of phyllosphere fungi (Cordier et al., 2012).

In natural environments, microorganisms usually live within complex ecological networks rather than existing in isolation (Faust & Raes, 2012). Co-occurrence patterns, which describe how species within a community coexist, are omnipresent and are crucial for understanding the structure of microbial communities and for revealing the potential interactions between microbial taxa (Kay et al., 2018; Ma et al., 2016). Network analysis, which can identify and describe taxon co-occurrence patterns in large datasets, has proven to be a powerful method that has been recently applied for characterization of bacterial communities across a wide variety of habitats (Baldassano & Bassett, 2016); including soil (Barberan et al., 2012; Jiao et al., 2016), ocean (Milici et al., 2016), gut (Liggenstoffer et al., 2010; Zhang et al., 2018b; Zhang et al., 2014), activated sludge (Ju et al., 2014), and mammalian milk (Li et al., 2017). These studies have uncovered previously unknown co-occurrence patterns, thereby offering new insight into the distribution of species associations. Several processes may be responsible for inter-taxa associations including niche differentiation, shared environmental requirements and phylogenetic relatedness (Kay et al., 2018; Zhang et al., 2018a). Relative to bacterial communities, there have been far fewer studies conducted using high throughput sequencing data to detect the co-occurrence patterns of fungal communities. By analyzing community composition of arbuscular mycorrhizal fungi across Europe, Bouffaud et al. (2016) suggested that closely-related taxa are more likely to co-occur than distantly-related species. Moreover, He et al. (2017) found greater linkage in soil fungal networks when the seasonal precipitation was manipulated, suggesting that inter-species interactions may be intensified to adapt to differences in water availability. In addition, a study conducted on the networks of fungal communities in soils where there has been a prolonged potato monoculture demonstrated scale-free, small world and modular properties (Lu et al., 2013). Scale-free network usually share an important property: a few fungi have a tremendous number of connections to other fungi, whereas most fungi have just a handful. Small world network usually showed small average path length and high clustering coefficient, which indicated that the fungus in this community has close interaction. Modularity is one measure of network structure. Networks with high modularity have dense connections between the nodes within modules but sparse connections between nodes in different modules.

Phyllosphere fungi, which occupy leaves superficially or intracellularly without causing visible symptoms, are important factors affecting plant health and ecosystem function (Drake, White Jr & Belanger, 2018; Gomes et al., 2018; Jumpponen & Jones, 2009). For example, Phyllosphere fungi can protect host plants from pathogen damage (Arnold et al., 2003; Mejia et al., 2008), enhance tolerances against herbivores (Albrectsen et al., 2010; Estrada, Wcislo & Van Bael, 2013; Hartley & Gange, 2009), and play an essential role in the initial decomposition of leaves following senescence (Guerreiro et al., 2018; Kembel & Mueller, 2014; Voriskova & Baldrian, 2013). Although the elevational distribution patterns of phyllosphere fungal communities have been previously described in the Gave Valley of the French Pyrenees (Cordier et al., 2012), such an examination of the elevational co-occurrence patterns of phyllosphere fungi in a subtropical climate has not yet been performed.

*Mussaenda shikokiana* Makino is a mostly autogamous plant species that is widely distributed throughout the warmer parts of China and Japan (Chen, Luo & Zhang, 2013). The floral diversity and pollination biology of *M. shikokiana* has been described in a previous study (Chen, Luo & Zhang, 2013), but little is known about the community structure of its phyllosphere fungal assemblages. To assess this, we used Illumina MiSeq sequencing data of fungal ribosomal internal transcribed spacer 2 (ITS2) to analyze the changes in the composition and inter-taxa interactions of the phyllosphere fungal communities inhabiting *M. shikokiana* along an altitudinal gradient in a subtropical forest. Specifically, we tried to address the following questions: (1) How does the richness and community composition of the phyllosphere fungal assemblage change in response to the elevation gradient? (2) Does the network topology of the fungal community shift significantly with elevation?

## Materials and Methods

### Study site and sampling

This study was conducted in the Laoshan Nature Reserve of Jingxiu, which is located in Guangxi province, China. This region possesses a subtropical moist climate and abundant vegetation. The annual mean rainfall is 1,824 mm, and the mean annual temperature is 17.0 °C with the lowest and highest temperatures in January (13.8 °C) and July (21.7 °C), respectively. Here, *M. shikokiana,* an erect shrub that possesses yellow hermaphrodite flowers and thinly papery leaves, occurs at the southern margin of its range at a mean altitude of 1000 m above sea level (Chen, Luo & Zhang, 2013; Chen, Luo & Zhang, 2014). The leaves of *M. shikokiana* contains several biologically active compounds that significantly stimulate immune activity and has been used as a Chinese folk medicine (Takeda, Nishimura & Inouye, 1977). Sampling was conducted at four different altitudinal stands (838 m, 934 m, 1,078 m and 1,185 m), each of which contained a high proportion of this species. Mean annual temperature (MAT) and mean annual precipitation (MAP) for each site were obtained from the WorldClim global climate data set with a resolution of 30 s (http://worldclim.org/version2; Table S1). At each elevation, we selected five individuals (>10 m apart from each other) that were in the fruiting stage (September; 2014). Nine mature and asymptomatic leaves from the middle of three current-year shoots were randomly collected from each individual plant, and immediately placed in sterile plastic bags containing sufficient silica gel for quick drying. The samples were then brought back to the laboratory and stored at −80 °C until DNA extraction.

### Illumina MiSeq sequencing preparation

Homogenization and mechanical cell disruption of the freeze-dried samples were carried out by grinding with liquid nitrogen using sterilized mortars and pestles. The resulting powder was then transferred to tubes with CTAB (2% CTAB, 100 mM Tris–HCl, 1.4 M NaCl, 20 mM EDTA, pH 8.0) extraction buffer preheated to 68 °C. DNA extraction was performed following the protocol modified by Cota-Sanchez, ReMarchuk & Ubayasena (2006). Briefly, an equal volume of chloroform–isoamylol mixture was added to each tube and mixed thoroughly to form an emulsion. The mixture was spun at 13,000 g for 12 min and the aqueous phase was removed into a fresh centrifuge tube. The aqueous phase containing DNA was re-extracted using chloroform:isoamyl alcohol (24:1) until no interface was visible. DNA was then precipitated with an equal volume of isopropanol. The resulting genomic DNA was precipitated at 10,000 g for 3 min and the DNA pellet was washed with 70% ethanol three times. Finally, the DNA pellet was resuspended in 100 µL TE buffer. A NanoDrop spectrophotometer (Thermo Fisher Scientific, Waltham, MA, USA) was used to quantify the extracted DNA.

To amplify the fungal ITS2 region of the nuclear ribosomal repeat unit, a semi-nested PCR approach was employed. Initial amplification of the entire ITS region was accomplished with conventional primers ITS1F (CTTGGTCATTTAGAGGAAGTAA) (Gardes & Bruns, 1993) and ITS4 (TCCTCCGCTTATTGATATGC) (White et al., 1990). The first round of amplification was carried out in 25 µL reaction volumes containing final concentrations of 0.2 mM dNTP, 0.4 µM of each primer, 2.5 mM MgSO_4_, 5 ng template DNA and 1 unit KOD-Plus-Neo DNA polymerase (TOYOBO, Osaka, Japan). Thermal cycling conditions for this amplification were as follows: 30 cycles of 94 °C (1 min), 56 °C (50 s), and 68 °C (1 min) after an initial denaturation at 94 °C (4 min), and followed by a final extension at 68 °C for 10 min. The PCR products were diluted 50 times and 1 µL of each dilution was used as the template for the second amplification of the fungal ITS2 region. The primer fITS7 (GTGARTCATCGAATCTTTG) (Ihrmark et al., 2012) and reverse primer ITS4 extended with a unique sample identification barcode were used for the second stage of PCR reaction, which was performed under the same amplification conditions. Negative PCR controls (in which the template DNA was replaced with sterile water) were used to evaluate whether there was contamination during the entire extraction and PCR process. Each sample was amplified in triplicate to minimize PCR biases and the three replicate products were mixed in equal volumes into a general sample that was purified by using an Ultra-Clean PCR cleanup kit (Mo Bio, Carlsbad, CA, USA). The samples were pooled with an equal molar amount (100 ng) from each sample and adjusted to 10 ng µl^−1^. The library was applied to an Illumina MiSeq PE250 platform for sequencing using the paired end ( 2 × 250 bp) option.

### Bioinformatics workflow

The trimming, sorting and quality filtering of reads were processed using the QIIME Pipeline (Caporaso et al., 2010). All sequences were demultiplexed into samples based on unique identification barcodes. FLASH-1.2.8 software (Magoc & Salzberg, 2011) was used to combine overlapping paired-end reads. The ITS2 region from the remaining sequences was identified and extracted using the fungal ITSx software (Bengtsson-Palme et al., 2013). Chimeras were detected and eliminated using the UCHIME program (Edgar et al., 2011) referenced with the UNITE database ( https://unite.ut.ee/; (Koljalg et al., 2013). Non-chimera sequences that passed the filtering processes were binned into operational taxonomic units (OTUs) at a 97% similarity cutoff using the UPARSE pipeline (Edgar et al., 2011). All singletons OTU were excluded from the dataset. Subsequently, the taxonomic identity of each OTU was determined based on BLAST search against the UNITE reference database, and a BLAST e-value <1e−was a criterion for matching. Further, OTUs occurring in only one sample and OTUs with <10 reads were removed because those rare sequences may have been derived from contaminations or PCR/sequencing errors (Toju, Tanabe & Ishii, 2016). For downstream analyses, each sample was rarefied to the minimum sequencing depth by randomly selecting subsets of reads implemented in the MOTHUR program (Schloss et al., 2009).

### Community analyses

To evaluate the comprehensiveness of the sampling strategy, the taxa accumulation curves for each site were calculated by employing the “specaccum” function in the vegan R package (Harrison et al., 2016; Oksanen et al., 2016). Vectors representing horizontal spatial structure were obtained by an analysis of principal coordinates of neighbor matrices (PCNM) (Borcard & Legendre, 2002) based on the geographical coordinates using the “PCNM” package (Legendre et al., 2010). Linear regression models were used to test the relationship between fungal OTU richness and elevation.

To identify the effects of elevation, PCNM vectors, and climate factors on phyllosphere fungal OTU richness, we first constructed generalized linear mixed models (GLMM) included elevation site as a random effect. However, we found that random factors explaining no variation in the data, so we switched to construct generalized linear models (GLM). These models were then examined by analysis of variance (ANOVA) tests. Moreover, the effects of those factors on fungal community structure was assessed by permutational multivariate analysis of variance (PERMANOVA) with site as the random effect using the vegan R package (9999 random permutations). Non-metric multidimensional scaling (NMDS) was performed with the Bray–Curtis distance based on a Hellinger-transformed matrix. Subsequently, the “envfit” function of the vegan R package was used to fit the elevation variable into the NMDS plot. Altitudinal distances, geographical distance and Bray-Curtis dissimilarity matrices were analyzed for correlations to evaluate the distance-decay rates, and Mantel tests were employed to assess the significance of the distance-decay regressions from zero (9999 random permutations) (Oono, Rasmussen & Lefevre, 2017). The appropriate trophic categories (symbiotroph, pathotroph and saprotroph) of each OTU were inferred by searching against the FUNGuild data base (Nguyen et al., 2016b).

To further investigate the phyllosphere fungal taxonomic distribution, significant taxonomic differences between the four elevation sites were identified using Linear Discriminant Analysis (LDA) effect size (LEfSe) (Segata et al., 2011), which introduces the non-parametric factorial Kruskal–Wallis (KW) sum-rank test (*α* = 0.05) to identify taxa having significant differential abundances between four elevation stands (employing one-against-all comparisons) (Bokulich et al., 2014). A logarithmic LDA score of 3.0 was set as the threshold for discriminative features.

To identify the indicator OTUs for each elevation site, LEfSe analysis and Legendre’s INDVAL analysis were combined as per Hubbard et al. (2018). Legendre’s INDVAL procedure was performed in R using the “labdsv” package (Sapkota et al., 2015). The fungal OTUs with an indicator value >0.25 and a significant *P* values (*P* < 0.05) were considered indicator species (Dufrene & Legendre, 1997; Sapkota et al., 2015). However, OTUs with low abundance hold little indicator information (Rime et al., 2015). To avoid random effects caused by rare OTUs, only those with >500 reads were defined as true key players (Eusemann et al., 2016).

### Network analyses

To remove poorly represented OTUs and reduce network complexity, only abundant OTUs with a proportion of the total sequences over 0.02% were kept in the OTU table. The co-occurrence network was inferred based on the Spearman’s Rho between the pairwise OTUs matrix constructed by the psych R package (Revelle, 2017). The *P*-values for multiple testing were calculated by using the false discovery rate (FDR) controlling procedure as per Benjamini & Hochberg (1995). A valid co-occurrence event was considered to be robust if the correlation coefficient *r* >—0.6— and if it was statistically significant at *P* < 0.05 (Widder et al., 2014). Network images for each elevation site were generated with Gephi (Bastian, Heymann & Jacomy, 2009). To describe the topology properties of the co-occurrence networks, the feature set (number of positive correlations, number of negative correlations, number of vertices, average degree, average path length, centralization betweenness, diameter, centralization degree and clustering coefficient) was calculated with the igraph R package (Csardi, 2006). Network diameter is a measure of how integrated the network is (Proulx, Promislow & Phillips, 2005). A network that has multiple fractured components would have a smaller average diameter (Lawes et al., 2017). Spearman correlations and significance tests (level at *p* < 0.05) between those properties were calculated and plotted using the corrplot R package (Taiyun & Viliam, 2017).

## Results

### Data characteristics

**Figure 1:**
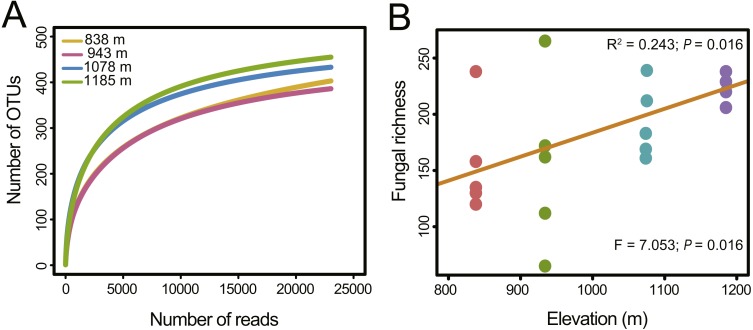
Fungal richness along the elevation gradient. (A) OTU accumulation curves of the phyllosphere fungal communities at four elevation sites. (B) OTU richness of phyllosphere fungal communities at each site. The results from the linear regressions and the one-way analysis of variance (ANOVA) are indicated in the upper and lower part of the figure, respectively.

**Figure 2:**
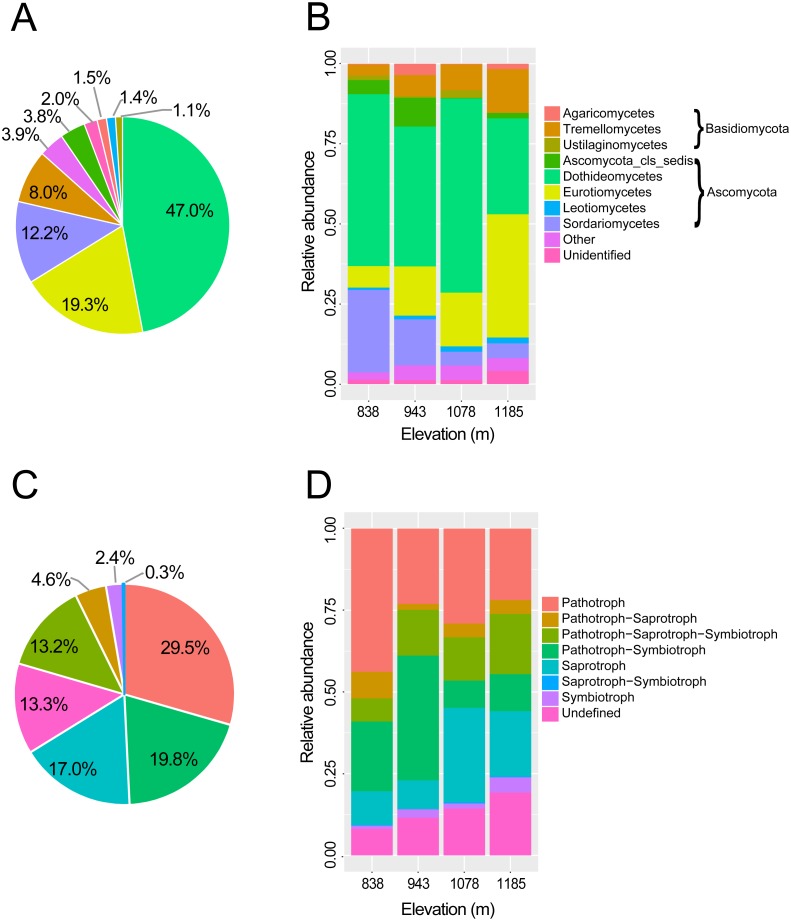
Taxonomic composition and trophic guilds of the fungal communities. Pie charts showing the distribution of sequences of (A) fungal classes and (C) fungal trophic guilds. Bar charts showing the taxonomic composition of the phyllosphere fungal communities in four elevation sites at (B) taxonomic class level and (D) tropic guilds. The fungal classes with a relative abundance of <0.1% were classified to “other”.

In total, 451,726 raw reads were obtained using Illumina MiSeq sequencing of the ITS2 amplicon libraries. After a series of filterings, 256,258 high-quality reads were clustered into 792 operational taxonomic units (OTUs) at a 97% sequence similarity cutoff. Finally, the sequence number of each sample was rarefied to 4606, resulting in a normalized dataset that contained 708 fungal OTUs. The abundance distribution of the OTUs was highly skewed, with plentiful rare and only a small number of very abundant OTUs. The 50 most abundant OTUs accounted for 72.4% of all sequences. The rarefaction and species accumulation curves reached a saturation plateau, indicating that our sampling and sequencing depths were sufficient to capture the majority of the phyllosphere fungal OTUs at each elevation site (Fig. 1A). Fungal communities were strongly dominated by members of the phylum Ascomycota (84.1% of all reads), followed by Basidiomycota (13.9%). The relative abundance of Basidiomycota increased slightly with elevation. At the class level, the most abundant OTUs were assigned to Dothideomycetes (47.0%), Eurotiomycetes (19.3%), Sordariomycetes (12.3%) and Tremellomycetes (8.0%) (Fig. 2A). Different community compositions between elevations were observed at this taxonomic level (Fig. 2B). For example, the abundance of the Eurotiomycetes and Tremellomycetes was higher at the highest elevation than at other sites, while Ustilaginomycetes species were hardly found at 1,185 m. Among the 52 identified orders, the three most abundant were Capnodiales (27.3%), Chaetothyriales (19.3%) and Pleosporales (11.9%). A total of 210 genera were classified, and *Cladosporium* (15.4%) was the most abundant genus in the *M. shikokiana* phyllosphere fungal community. The taxonomic assignment of each OTU is provided in Table S2.

At the genus level, 71.9% of OTUs (509 of 708) were assigned to functional groups in FUNGuild database (Nguyen et al., 2016b). Dominant guilds were classified in seven groups of trophic modes (Fig. 2B). Pathotrophic fungi were the highest in abundance (29.5% of all sequences), followed by the pathotroph-symbiotroph group (19.8%), and the saprotroph group (17.0%; Fig. 2C). Compositions of trophic guilds were also different between elevations (Fig. 2D). The relative abundance of the pathotrophs was highest at the lowest elevation, while the symbiotrophs were more abundant at the highest elevation than at other sites.

### Variation in fungal richness and community composition

**Figure 3:**
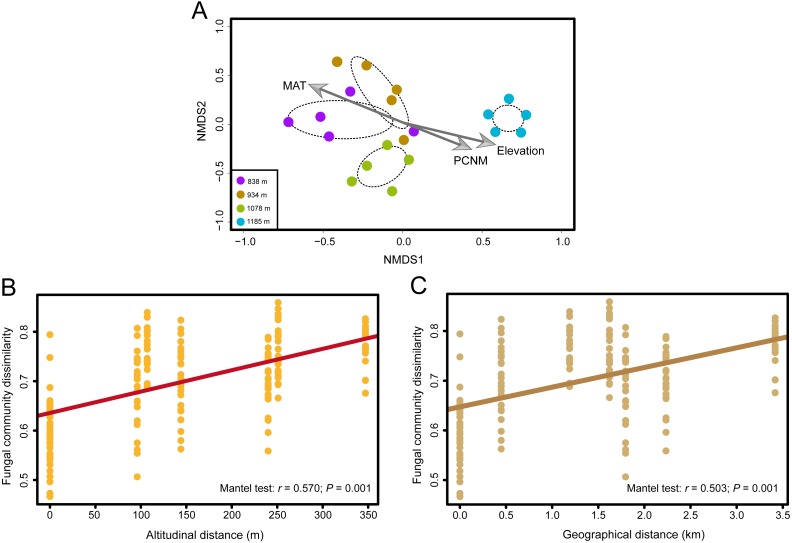
The effects of elevation gradient, geographical distance and climate factor on the fungal community composition. (A) Nonmetric multidimensional scaling (NMDS) ordination of variation in community structure (Bray–Curtis dissimilarity) of the phyllosphere fungal assemblages. The arrow represents significant (*P* < 0.01) vectors chosen by the “envfit” function to fit the fungal communities. (B) Correlations between altitudinal distance and Bray–Curtis dissimilarities of the phyllosphere fungal community. (C) Correlations between geographical distance and Bray-Curtis dissimilarities of the phyllosphere fungal community. Mantel test results are shown on the figures.

Fungal OTU richness increased significantly with increasing elevation (*R*^2^ = 0.243, *P* = 0.016; Fig. 1B). The generalized linear model (GLM) also supported a significant effect of elevation on *M. shikokiana* phyllosphere fungal richness (*F* = 6.484, *P* = 0.021; Table S3). The nonmetric multidimensional scaling (NMDS) plot revealed a general clustering according to elevation site, and the members of the 1,185 m stand were clearly separated from the other clusters (Fig. 3A). The fit of the variable selected by the “envfit” function showed that patterns of community similarity were strongly structured by elevation (*R*^2^ = 0.736, *P* = 0.001) followed by PCNM vector (*R*^2^ = 0.534, *P* = 0.003) and MAT (*R*^2^ = 0.739, *P* = 0.001). The significant effects of those factors on the community structure were further reflected in the results by the permutational multivariate analysis of variance (PERMANOVA), as ∼16.0%, 12.2% and 10.9% of community variation could be explained by elevation, PCNM and MAT, respectively (Table 1). Furthermore, Mantel tests indicated that fungal community dissimilarities were significantly positively correlated with altitudinal distance (*r* = 0.570, *P* = 0.001; Fig. 3B) and geographical distance (*r* = 0.503, *P* = 0.001; Fig. 3C), respectively.

**Table 1:** Relative importance of elevation and geographical distance on the phyllosphere fungal community composition as revealed by permutational multivariate analysis of variance (PERMANOVA).

Variable	DF	SS	F-statistics	R^2^	*P* value
Elevation	1	0.765	4.218	0.160	^***^
PCNM	1	0.584	3.219	0.122	^***^
MAT	1	0.519	2.861	0.109	^***^
Residuals	16	2.900		0.608	
Total	19	4.767		1.000	

**Notes.**

DF degrees of freedom SS sum of squares PCNM principal coordinates of neighbor matrices MAT mean annual temperature

*** *P* < 0.001.

**Figure 4:**
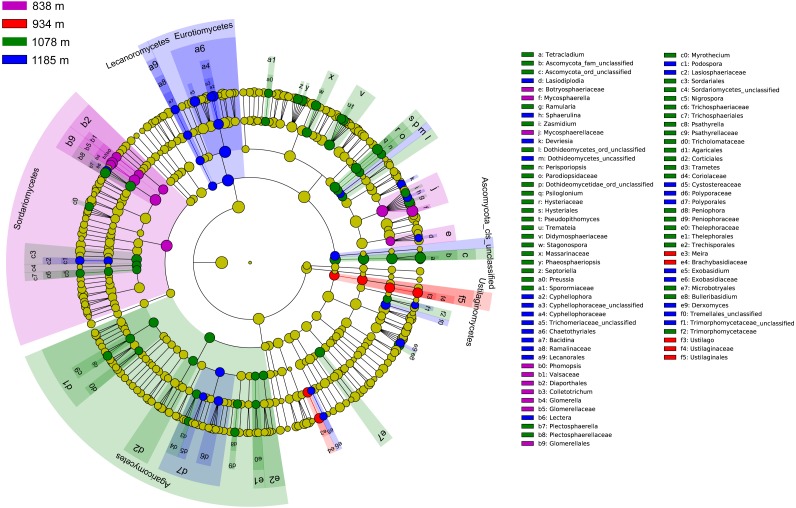
Cladogram showing significant discriminative taxa of the phyllosphere fungi associated with *Mussaenda shikokiana* along the elevation gradient. The filled circles from inside to out indicate the taxonomic levels with phylum, class, order, family and genus. Circles or nodes shown in color corresponding to that of different elevation sites represented a significantly more abundant group.

The community compositions of phyllosphere fungi between the four elevation sites were significantly different, as is shown by the linear discriminant analysis effect size (LEfSe) cladogram (Fig. 4). These results indicate that many groups at different taxonomic levels can be significantly distinguished along the elevation gradient. For example, Sordariomycetes, Ustilaginomycetes, and Agaricomycetes appeared as main discriminant clades at 838 m, 934 m, and 1,078 m, respectively. Eurotiomycetes and Lecanoromycetes are the most differentially abundant fungal classes at 1,185 m. At the taxonomic order level, Glomerellales, Diaporthales and Chaetothyriales were significantly enriched at 838 m; Ustilaginales was found to predominate at 934 m; Hysteriales, Sordariales, Microbotryales, Agaricales, Corticiales and Thelephorales were more abundant at 1,078 m; and Chaetothyriales, Polyporales and Lecanorales presented differential abundances at 1,185 m.

**Figure 5:**
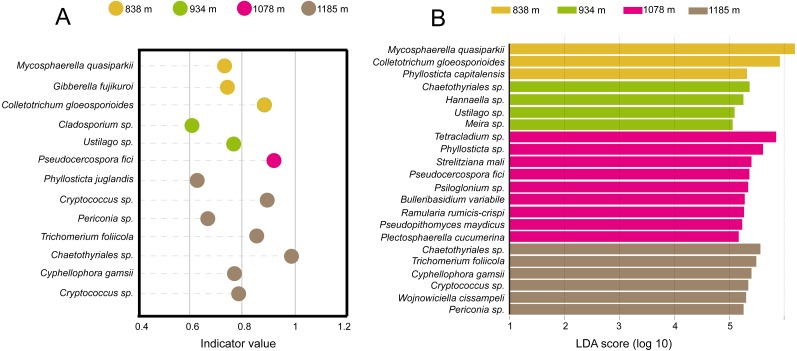
Identification of indicator species based on (A) INDVAL analysis and (B) LEfse analyses. Indicator fungi with INDVAL indicator value >0.25 or LDA scores >3 at four elevation sites.

Taken together, the LEfSe and INDVAL analyses detected a total of four indicator OTUs (*Mycosphaerella quasiparkii*, *Gibberella fujikuroi*, *Colletotrichum gloeosporioides* and *Phyllosticta capitalensis*) that were significantly associated with the phyllosphere at 838 m; five indicator OTUs (*Ustilago sp.*, *Cladosporium sp.*, *Meira sp. Hannaella sp.* and *Chaetothyriales sp.*) at 934 m; nine indicator OTUs (*Plectosphaerella cucumerina, Pseudopithomyces maydicus*, *Ramularia rumicis-crispi*, *Bulleribasidium variabile*, *Psiloglonium sp.*, *Pseudocercospora fici*, *Strelitziana mali*, *Phyllosticta sp.* and *Tetracladium sp.*) at 1,078 m; and eight indicator OTUs (*Cryptococcus sp.*, *Cyphellophora gamsii*, *Chaetothyriales sp.*, *Trichomerium foliicola*, *Periconia sp.*, *Cryptococcus sp.*, *Phyllosticta juglandis* and *Wojnowiciella cissampeli*) at 1,185 m (Fig. 5).

### Key topological properties of the co-occurrence networks

**Figure 6:**
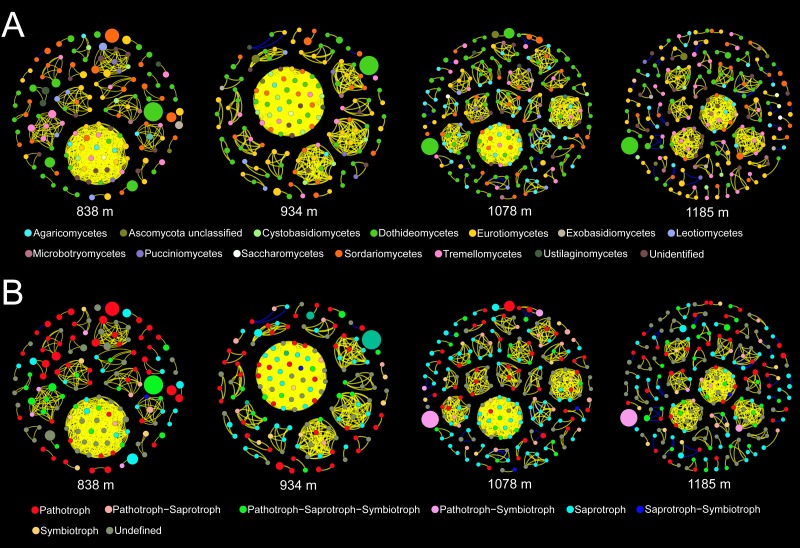
Co-occurrence networks for the phyllosphere fungal communities at each elevation site. The node color indicates the corresponding taxonomic assignment at class level (A) and trophic mode (B). The node sizes are proportional to the OTU abundances and the color of each line reflects positive (yellow) or negative (blue) associations.

**Figure 7:**
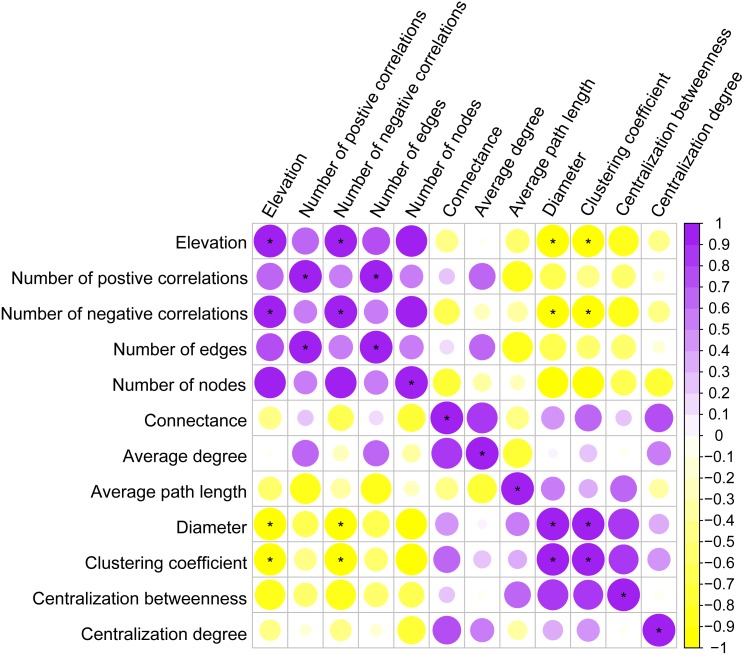
Spearman correlations between topological properties of the co-occurrence networks and elevation gradient. ∗ indicates *P* < 0.05.

Co-occurrence patterns of the phyllosphere fungal communities were constructed for each elevation site (Fig. 6). Statistically significant and strong correlations were visualized as fungal association networks. Topological properties delineated the interaction pattern nodes, and were used to distinguish differences between the network structures of the four elevation groups. The clustering coefficient, which reflects how well nodes are connected with neighboring ones, decreased significantly with increasing elevation (Fig. 7). This indicated that the network became more discrete and sparser with increasing elevation. The network diameters of the *M. shikokiana* phyllosphere fungal communities showed a significantly decrease with elevation (Fig. 7). In total, over 98% of all correlations were positive. However, the negative links were significantly positive related to elevation (Fig. 7), indicating an increasing mutual exclusion between the fungal species.

## Discussion

### Overall taxonomic diversity and community richness

A taxonomically diverse assemblage of phyllosphere fungi (708 OTUs) was detected in *M. shikokiana,* with taxa from numerous genera (210) and families (134). The OTU richness was higher than that of the needle mycobiome of *Picea glauca* at the northern treeline in Alaska (Sapkota et al., 2015), but lower than that of the phyllosphere fungal community of *Fagus sylvatica* in the French Pyrenees (Cordier et al., 2012). The phyllosphere fungal communities were largely composed of Ascomycota and dominated by members of Dothideomycetes, as previously reported in foliar fungal studies. For example, Yang et al. (2016a) found that the phyllosphere fungi of *Betula ermanii* in the Changbai mountains were dominated by Ascomycota, and in particular by members of Dothidiomycetes. Harrison et al. (2016) also revealed that the majority of fungal OTUs associated with the leaves of *Sequoia sempervirens* in the western United States were assigned to the Dothideomycetes. Similar observations have been documented in the foliar fungal communities of *Eucalyptus grandis* (Kemler et al., 2013), *Picea glauca* (Eusemann et al., 2016), *Picea abies* (Nguyen et al., 2016a), *Metrosideros ploymorpha* (Zimmerman & Vitousek, 2012) and *Hopea ferrea* (Izuno et al., 2016). However, different dominant taxa were detected in *Fagus sylvatica* (Cordier et al., 2012; Siddique & Unterseher, 2016; Unterseher et al., 2016), and several bryophytes species (Davey et al., 2013), indicating different environmental filtration and host selection across distinct geographic origins and plant species (Yang et al., 2016a; Peršoh, 2015; Rodriguez et al., 2009)).

Most fungal taxa identified here could be assigned to different functional guilds. The seven main ecological guilds (pathotroph, symbiotroph, saprotroph, pathotroph-saprotroph, pathotroph-symbiotroph, saprotroph-symbiotroph and saprotroph-pathotroph-saprotroph) were all found in our study. Many fungal species belong to more than one of the ecological categories, which is very common in fungal kingdom. For example, *Fusarium culmorum* could be a plant pathogen causing crown rot in cereals (Hollaway et al., 2013), but also could be a symbiotic fungal endophyte conferring salt tolerance to *Leymus mollis* (Rodriguez et al., 2008). Our result is consistent with a recent study on the fungal endophytes of *Fagus sylvatica*, and suggest undeniable advantages of high-throughput sequencing over traditional cultivation in terms of uncovering a good representation of the major functional guilds as well as rare fungal taxa (Siddique, Khokon & Unterseher, 2017). In addition to saprotrophic and symbiotrophic taxa, numerous pathotrophic fungi (29.5%) were detected on *M. shikokiana* leaves. Several indicator species, including *Gibberella fujikuroi*, *Ramularia rumicis-crispi*, *Mycosphaerella quasiparkii*, *Plectosphaerella cucumerina* and *Strelitziana mali* also have been recorded as fungal phytopathogens (De Vos et al., 2014; Petriacq, Stassen & Ton, 2016; Wardle & Lindahl, 2014). In accordance with this, Sapkota et al. (2015) found that host-specific pathogens lived in a “sea” of nonspecific fungi in the phyllosphere of six Poaceae species. Notably, several endophytic fungi may be latent pathogens (Vacher et al., 2016). *Colletotrichum gloeosporioides*, a significant indicator at 838 m, has previously been reported as an endophytic and symbiotic fungus that serves as a biocontrol agent for *Theobroma cacao* pathogens (Mejia et al., 2008), and is itself also an important plant pathogen causing anthracnose (Phoulivong et al., 2010). Alvarez-Loayza et al. (2011) found that high light environments can trigger the production of H_2_O_2_ by *Diplodia mutila*, a common asymptomatic endophyte in *Iriartea deltoidea*, resulting in pathogenicity due to constraining niche-space filling of the plant species.

### Effects of elevation on fungal richness and community composition

Our results show that phyllosphere fungal richness and the community structure of fungi associated with *M. shikokiana* are affected significantly by elevation. These findings agree with the patterns of fungal endophytes of *B. ermanii* in the Changbai mountains (Yang et al., 2016b) and of *F. sylvatica* phyllosphere fungi in the French Pyrenees (Cordier et al., 2012). Shifts in community composition along the elevational gradient were significantly related to changes in temperature. Many studies showed that altitudinal distribution patterns of fungal assemblages often associate with climatic variables and environmental factors that co-vary with elevation. The variations in incidence and the abundance of *F. sylvatica* phyllosphere fungi were significantly correlated with variation in average temperature (Cordier et al., 2012). The endophytic community composition of *M. ploymorpha* can be driven by rainfall along elevation gradient (Zimmerman & Vitousek, 2012). The ectomycorrhizal fungal richness on Mount Kinabalu in Malaysia is highest in the elevation zone with the most available water (Geml et al., 2017). Jarvis, Woodward & Taylor (2015) also suggested that significant climatic niche partitioning between ectomycorrhizal fungi is associated with a single host along a short (300 m) elevation gradient. Therefore, variation in some climatic parameters that we did not record in this study—such as humidity, wind exposure or UV radiation along the elevation gradient—may contribute to the distribution patterns of phyllosphere fungi in *M. shikokiana*. The indicator species at the elevation of 1,185 m include the yeasts *Cryptococcus spp*. and the melanized black yeast *Cyphellophora gamsii*, all of which possess a strong ability to withstand UV radiation (Sapkota et al., 2015), which corresponds to the fact that solar radiation is higher at higher elevations. In addition to the importance of elevation site parameters in shaping fungal communities, abundant research has suggested a significant interplay between phyllosphere fungi and their host plants (Eusemann et al., 2016). Whipps et al. (2008) summarized that the development of phyllosphere microbiota must become involved in the phenotypic traits (leaf chemistry, physiology or morphology) exhibited by host plants. For instance, elevated leaf carbon availability could significantly enhance the phyllosphere fungal richness of *B. ermanii* along the elevation gradient due to broadening of niche space associated with the phyllosphere environment (Yang et al., 2016b). Furthermore, some phyllosphere fungi are dormant saprotrophs involved in the decomposition of senescent leaves (Vacher et al., 2016). The significant indicator species at the highest elevation in our study, *Cyphellophora gamsii*, is a saprotrophic fungus usually found in leaf litter (Crous et al., 2016; Madrid et al., 2016). Leaf litter at higher elevations is usually more recalcitrant because of increased nutrient limitation and leaf thickness; these features may facilitate the presence of specialized fungi capable of breaking down recalcitrant organic matter (Looby, Maltz & Treseder, 2016). This pattern further supports a greater richness of fungi released to the soil and air through decomposition, which may lead to increases in phyllosphere fungal richness by horizontal transmission (Osono & Mori, 2003). In addition to altitudinal distance, geographical distance also plays an important role in shaping phyllosphere fungal community composition, indicating dispersal limitation or environment isolation over a local scale (Hanson et al., 2012).

### Co-occurrence networks of phyllosphere fungi

By providing a vivid and simplified version of the complex ecological interactions that shape microbial communities, network analyses of the co-occurrence patterns of significant taxa may help to deepen our understanding of the altitudinal distribution patterns of phyllosphere fungal assemblages along elevation gradients (Eiler, Heinrich & Bertilsson, 2011). Our results showed that the phyllosphere fungal network of *M. shikokiana* shifted between different elevation sites. Most of the correlations between fungal OTUs (838 m: 99%, 934 m: 99%, 1,078 m: 98%, and 1,185 m: 97%) were identified as co-occurrences (positive correlations). Dominant positive associations suggest that most fungal taxa may synergistically act or share similar ecological niches in the phyllosphere environment (Zhang et al., 2018a). A prevalence of positive correlations has also been found in other microbial networks (Aschenbrenner et al., 2017; Chow et al., 2014), although a much lower proportion was found in soil fungal communities. It is possible that nutrient use efficiency and mechanisms underlying fungal community assembly in soil are markedly different from those in the phyllosphere (Peršoh et al., 2018).

Although negative correlations, which suggest co-exclusion between two taxa, were much rarer than positive ones, the number of negative links increased significantly with elevation. This trend may indicate a more competitive relationship between phyllosphere fungi at higher elevations. In phyllosphere microbial communities, competition for limited space and nutrient resources, as well as the production of metabolic compounds and interference with hormone signaling pathways, are the major mechanisms by which microbial inhabitants inhibit each other (Vorholt, 2012). This result agrees with the finding that networks of endophytic communities are almost unconnected in more northern, temperate regions (Guerreiro et al., 2018). Interestingly, many negative interactions occurred between pathogenic members and other taxa; a similar finding has been reported in endophytic fungal communities (Peršoh, 2013). This may help to maintain community stability and plant health. Fokkema (1993) suggested that competition between pathogens and saprotrophs acts as a natural occurring biocontrol process in the phyllosphere. This complex microbial interaction might possess barrier effects enhancing the plant’s resistance to pathogenic infections in the phyllosphere (Vorholt, 2012). Negative correlations can finally result in a less stable network structure (Lawes et al., 2017). This increasing tendency of negative correlations also could be partly responsible for less coherent and compact network structure at higher elevation, as shown by clustering coefficients and diameters (Figs. 6 and 7). Lower elevations may provide more favorable conditions for fungal interaction and niche sharing. Higher elevations are often associated with harsher environmental conditions for plant growth, such as stronger ultraviolet exposure and lower temperatures (Pauchard et al., 2009). The decrease in connectivity and integrity of the co-occurrence networks may indicate a change towards stochastic assembly processes, prevention of biotic communication, or shifts in biogeochemical pathways due to environmental stress (Lawes et al., 2017).

## Conclusions

In summary, this study reports high-throughput sequencing data that suggests that an elevation gradient of only 350 m can entail significant shifts in OTU richness, community composition, and co-occurrence patterns of phyllosphere fungi in a single host species. The phyllosphere fungal assemblages inhabiting *M. shikokiana* show high diversity in taxonomic classification and trophic stages. Fungal richness showed a pattern of monotonal increase along an elevation gradient. In addition, elevation is a critical predictor of the fungal community structure. Shifts in community composition along an elevation gradient were clearly related to MAT, which is suggestive of significant temperature niche partitioning among phyllosphere fungal species. A distance-decay pattern was also found in the fungal assemblages. Co-occurrence networks became less centralized and more fractured with increasing elevation. However, limited information on abiotic and biotic factors that covary with elevation demands further investigation to determine the exact drivers and mechanisms of this altitudinal partitioning.

##  Supplemental Information

10.7717/peerj.5767/supp-1 Supplemental Information 1 Raw sequencing data of the five individuals at 838 m

10.7717/peerj.5767/supp-2 Supplemental Information 2 Raw sequencing data of the five samples at 934 m

10.7717/peerj.5767/supp-3 Supplemental Information 3 Raw sequencing data of the five individuals at 1,078 m

10.7717/peerj.5767/supp-4 Supplemental Information 4 Raw sequencing data of the five individuals at 1,185 m

10.7717/peerj.5767/supp-5 Table S1 Sampling details of *Mussaenda shikokiana*

10.7717/peerj.5767/supp-6 Table S2 The taxonomic assignment of each phyllosphere fungal OTU

10.7717/peerj.5767/supp-7 Table S3 Effect of elevation, geographical distance, and climate on the phyllosphere fungal richness using generalized linear models (GLM) DF, degrees of freedom, SS, sum of squares, PCNM, principal coordinates of neighbor matrices, and MAT: mean annual temperature. ^∗^ indicates *P* < 0.05.

## Additional Information and Declarations

### DNA Deposition

The following information was supplied regarding the deposition of DNA sequences: Representative sequences of the fungal OTUs are available in European Nucleotide Archive (ENA) under accession number: PRJEB25945.

### Data Availability

The following information was supplied regarding data availability: The raw data are provided in the Supplemental Files.
