# Supplementary material for: Shifts in community composition and co-occurrence patterns of phyllosphere fungi inhabiting Mussaenda shikokiana along an elevation gradient
Source: PeerJ. 2018 Oct 12;6:e5767. doi: 10.7717/peerj.5767 (PMC6187995; doi:10.7717/peerj.5767)
Supplement: Table S1 [file peerj-06-5767-s005.docx]

**Table S1 Sampling details of *Mussaenda shikokiana***

|  | Elevation (m) | Latitude (E) | Longitude (N) | MAT (℃) | MAP (mm) |
| --- | --- | --- | --- | --- | --- |
| Site 1 | 838 | 24.122 | 110.199 | 16.82 | 1682 |
| Site 2 | 934 | 24.108 | 110.208 | 16.57 | 1690 |
| Site 3 | 1078 | 24.104 | 110.209 | 15.89 | 1709 |
| Site 4 | 1185 | 24.095 | 110.215 | 15.52 | 1782 |
