# Supplementary material for: Shifts in community composition and co-occurrence patterns of phyllosphere fungi inhabiting Mussaenda shikokiana along an elevation gradient
Source: PeerJ. 2018 Oct 12;6:e5767. doi: 10.7717/peerj.5767 (PMC6187995; doi:10.7717/peerj.5767)
Supplement: Table S3 — DF, degrees of freedom, SS, sum of squares, PCNM, principal coordinates of neighbor matrices, and MAT: mean annual temperature. ∗ indicates P < 0.05. [file peerj-06-5767-s007.docx]

**Table S3** Effect of elevation, geographical distance, and climate on the phyllosphere fungal richness as assessed by generalized linear model (GLM)

| Variable | DF | SS | F value | *P* value |
| --- | --- | --- | --- | --- |
| Elevation | 1 | 14877 | 6.484 | 0.021* |
| PCNM | 1 | 1003 | 0.437 | 0.518 |
| MAT | 1 | 252 | 0.110 | 0.745 |
| Residuals | 16 | 36712 |  |  |

DF: degrees of freedom, SS: sum of squares, PCNM: principal coordinates of neighbor matrices, and MAT: mean annual temperature. * indicates P < 0.05.
